# Gene model for the ortholog of *Pi3K21B* in *Drosophila ananassae*

**DOI:** 10.17912/micropub.biology.001009

**Published:** 2026-08-26

**Authors:** Anne E. Backlund, Wyatt Brigham, Clare Dunne, James J. Youngblom, Stephanie Toering Peters, Norma Velazquez-Ulloa, Solomon Tin Chi Chak, Chinmay P. Rele, Laura Reed

**Affiliations:** 1 The University of Alabama, Tuscaloosa, AL USA; 2 California State University Stanislaus, Turlock, CA USA; 3 Wartburg College, Waverly, IA, USA; 4 Lewis and Clark College, Portland, OR USA; 5 SUNY Old Westbury, Old Westbury, NY USA

## Abstract

Gene model for the ortholog of *Phosphatidylinositol 3-kinase 21B* ( *Pi3K21B* ) in the May 2011 (Agencourt dana_caf1/DanaCAF1) Genome Assembly (GenBank Accession: GCA_000005115.1 ) of *Drosophila ananassae* . This ortholog was characterized as part of a developing dataset to study the evolution of the Insulin/insulin-like growth factor signaling pathway (IIS) across the genus *Drosophila* using the Genomics Education Partnership gene annotation protocol for Course-based Undergraduate Research Experiences.

**Figure 1. Genomic neighborhoods for Pi3K21B in Drosophila melanogaster and D. ananassae:** **(A) Synteny** [1] **comparison of the genomic neighborhoods for *Pi3K21B* in *Drosophila melanogaster* and *D. ananassae* .** Thin underlying arrows indicate the DNA strand within which the reference gene– *Pi3K21B* –is located in *D. melanogaster* (top) and *D. ananassae* (bottom). The thin arrow pointing to the right indicates that *Pi3K21B* is on the positive (+) strand in *D. melanogaster* , and the thin arrow pointing to the left indicates that *Pi3K21B* is on the negative (-) strand in *D. ananassae* . The wide gene arrows pointing in the same direction as *Pi3K21B* are on the same strand relative to the thin underlying arrows, while wide gene arrows pointing in the opposite direction of *Pi3K21B* are on the opposite strand relative to the thin underlying arrows. White gene arrows in *D. ananassae* indicate orthology to the corresponding gene in *D. melanogaster* . Gene symbols given in the *D. ananassae* gene arrows indicate the orthologous gene in *D. melanogaster* , while the locus identifiers are specific to *D. ananassae* . **(B) Gene Model in GEP UCSC Track Data Hub** (Raney et al., 2014). The coding-regions of *Pi3K21B* in *D. ananassae* are displayed in the User Supplied Track (black); coding CDSs are depicted by thick rectangles and introns by thin lines with arrows indicating the direction of transcription. Subsequent evidence tracks include BLAT Alignments of NCBI RefSeq Genes (dark blue, alignment of Ref-Seq genes for *D. ananassae* ), Spaln of *D. melanogaster* Proteins (purple, alignment of Ref-Seq proteins from *D. melanogaster* ), Transcripts and Coding Regions Predicted by TransDecoder (dark green), RNA-Seq from Adult Females, Adult Males, and *Wolbachia* -cured Embryos (red, light blue, and pink, respectively); alignment of Illumina RNA-Seq reads from *D. ananassae* ), and Splice Junctions Predicted by regtools using *D. ananassae* RNA-Seq ( SRP006203 , SRP007906 , PRJNA257286 , PRJNA388952 ). Splice junctions shown have a minimum read-depth of 10 with 50-99, 500-999, >1000 supporting reads in green, brown, and red, respectively. **(C) Dot Plot of Pi3K21B-PB in *D. melanogaster* ( *x* -axis) vs. the orthologous peptide in *D. ananassae* ( *y* -axis).** Amino acid number is indicated along the left and bottom; coding-CDS number is indicated along the top and right, and CDSs are also highlighted with alternating colors. Line breaks in the dot plot indicate mismatching amino acids at the specified location between species. **(D)** **Gene Model in GEP UCSC Track Data Hub** (Raney et al., 2014). The same evidence tracks as Figure 1B are shown in this image. In *D. melanogaster* , *Pi3K21B* -RC and *Pi3K21B* -RD have one CDS. However, in *D. ananassae* , these isoforms have two CDSs where the first CDS contains the most viable start codon for these proteins.

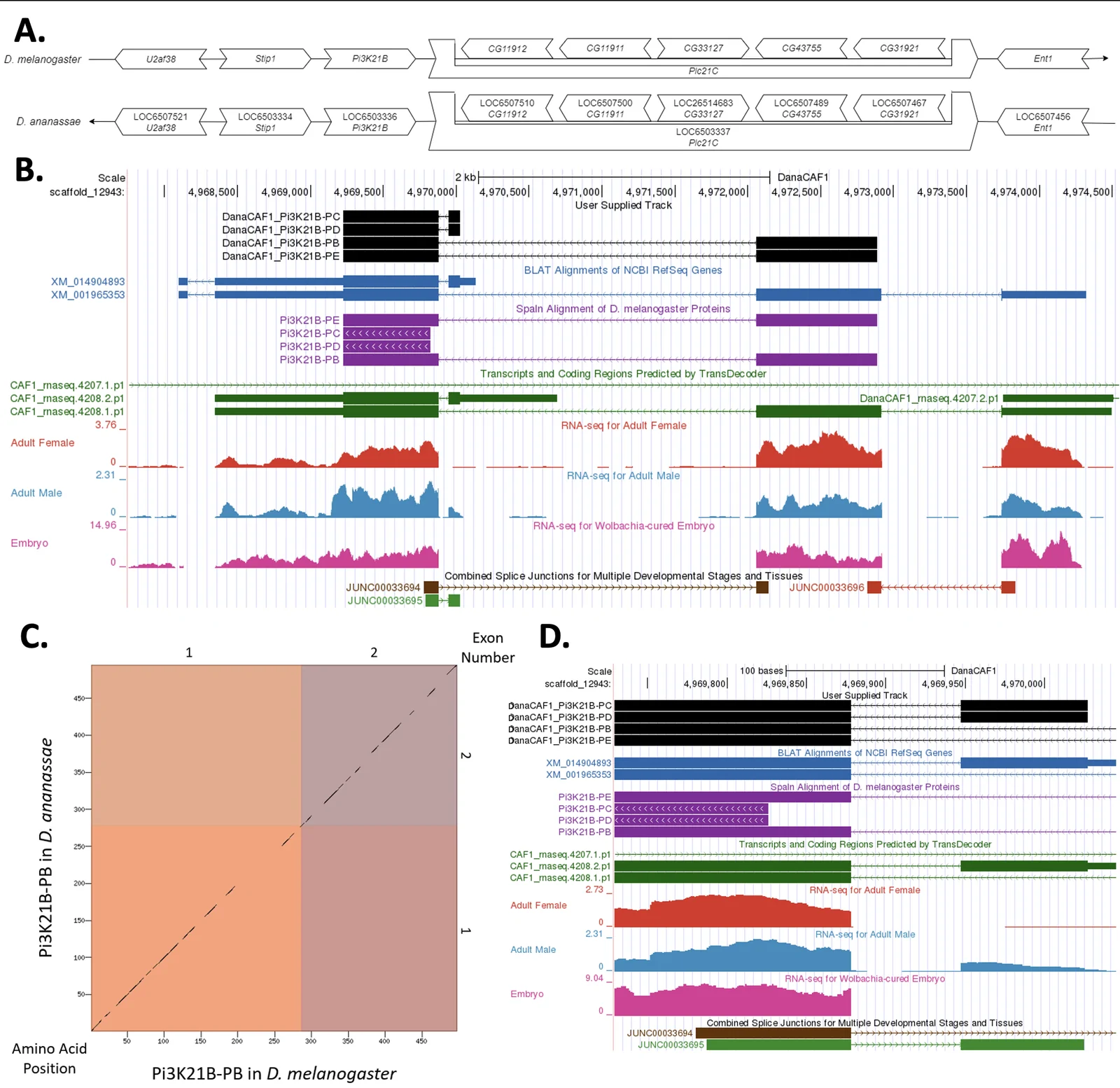



## Description

**Table:** 

* This article reports a predicted gene model generated by undergraduate work using a structured gene model annotation protocol defined by the Genomics Education Partnership (GEP; thegep.org ) for Course-based Undergraduate Research Experience (CURE). The following information in this box may be repeated in other articles submitted by participants using the same GEP CURE protocol for annotating Drosophila species orthologs of Drosophila melanogaster genes in the insulin signaling pathway. * "In this GEP CURE protocol students use web-based tools to manually annotate genes in non-model *Drosophila* species based on orthology to genes in the well-annotated model organism fruitfly *Drosophila melanogaster* . The GEP uses web-based tools to allow undergraduates to participate in course-based research by generating manual annotations of genes in non-model species (Rele et al., 2023). Computational-based gene predictions in any organism are often improved by careful manual annotation and curation, allowing for more accurate analyses of gene and genome evolution (Mudge and Harrow 2016; Tello-Ruiz et al., 2019). These models of orthologous genes across species, such as the one presented here, then provide a reliable basis for further evolutionary genomic analyses when made available to the scientific community.” (Myers et al., 2024). “The particular gene ortholog described here was characterized as part of a developing dataset to study the evolution of the Insulin/insulin-like growth factor signaling pathway (IIS) across the genus *Drosophila* . The Insulin/insulin-like growth factor signaling pathway (IIS) is a highly conserved signaling pathway in animals and is central to mediating organismal responses to nutrients (Hietakangas and Cohen 2009; Grewal 2009).” (Myers et al., 2024). “ *D* . *ananassae* (NCBI:txid7217) is part of the *melanogaster* species group within the subgenus *Sophophora* of the genus *Drosophila* (Sturtevant 1939; Bock and Wheeler 1972). It was first described by Doleschall (1858). *D. ananassae* is circumtropical (Markow and O'Grady 2005; https://www.taxodros.uzh.ch, accessed 1 Feb 2023), and often associated with human settlement (Singh 2010). It has been extensively studied as a model for its cytogenetic and genetic characteristics, and in experimental evolution (Kikkawa 1938; Singh and Yadav 2015).” (Lawson et al., 2024).

We propose a gene model for the *D. ananassae* ortholog of the *D. melanogaster* *Pi3K21B* ( *Pi3K21B* ) gene. The genomic region of the ortholog corresponds to the uncharacterized protein XP_ 001965389.2 (Locus ID LOC6503336 ) in the May 2011 (Agencourt dana_caf1/DanaCAF1; Drosophila 12 Genomes Consortium et al., 2007) Genome Assembly of *D. ananassae* ( GCA_000005115.1 ). This model is based on RNA-Seq data from *D. ananassae* ( SRP006203 , SRP007906 , PRJNA257286 , PRJNA388952 ; Graveley et al., 2011) and *Pi3K21B* in *D. melanogaster* using FlyBase release FB2023_03 ( GCA_000001215.4 ; Larkin et al., 2021; Gramates et al., 2022; Jenkins et al., 2022).

The gene product of the *Pi3K21B* gene (FBgn0020622), p60, was identified through affinity purification based on binding to a phosphorylated peptide in a heterodimer with p110, the Pi3K92E gene product (Weinkove et al., 1997). Additional experiments indicated that the p60:p110 complex was present in all life cycle stages in *Drosophila* , and demonstrated protein and lipid phosphatase activity of the complex *in vitro* (Weinkove et al., 1997). Null alleles were generated in *Drosophila* through P-element mobilization, and homozygous null animals display reduced larval growth with death in the third instar or early pupal stage of development (Weinkove et al., 1999). Further work indicated that the p60 adaptor protein was required for PI3K activity in the insulin signaling pathway, and was involved in determining both cellular and organismal size and growth (Britton et al., 2002; Oldham et al., 2002).

***Synteny***

The reference gene, *Pi3K21B* , occurs on chromosome 2L in *D. melanogaster* and is flanked upstream by *U2 small nuclear riboprotein auxiliary factor 38* ( *U2af38* ) and *Stress induced phosphoprotein 1* ( *Stip1* ) and downstream by *Phospholipase C at 21C* ( *Plc21C* ) and *Equilibrative nucleoside transporter 1* ( *Ent1* ). There are five genes nested within *Plc21C* : *CG11912 , CG11911 , CG33127 , CG43755* , and *CG31921* . The *tblastn* search of *D. melanogaster* *Pi3K21B* -PB (query) against the *D. ananassae* (GenBank Accession: GCA_000005115.1 ) Genome Assembly (database) placed the putative ortholog of *Pi3K21B* within scaffold_12943 at locus LOC6503336 ( XP_001965389.2 )— with an E-value of 3e-117 and a percent identity of 66.67%. Furthermore, the putative ortholog is flanked upstream by LOC6507521 ( XP_001965391.1 ) and LOC6503334 ( XP_001965390.1 ), which correspond to *U2af38* and *Stip1* in *D. melanogaster* (E-value: 1e-175 and 0.0; identity: 93.89% and 90.00%, respectively, as determined by *blastp* ; Figure 1A ; Altschul et al., 1990). The putative ortholog of *Pi3K21B* is flanked downstream by LOC6503337 ( XP_032311151.1 ) and LOC6507456 ( XP_001965382.1 ), which correspond to *Plc21C* and *Ent1* in *D. melanogaster* (E-value: 0.0 and 0.0; identity: 92.53% and 85.03%, respectively, as determined by *blastp* ). There are five genes nested within *Plc21C* : LOC6507510 ( XP_001965388.1 ), LOC6507500 ( XP_001965387.1 ), LOC26514683 ( XP_014760654.1 ), LOC6507489 ( XP_014760615.1 ), and LOC6507467 ( XP_001965384.1 ). These correspond to *CG11912 , CG11911 , CG33127 , CG43755* , and *CG31921* in *D. melanogaster* (E-value: 1e-111, 9e-169, 5e-163, 1e-128, 3e-153; percent identity: 58.58%, 77.98%, 81.99%, 57.06%, 42.24%). The putative ortholog assignment for *Pi3K21B* in *D. ananassae* is supported by the following evidence: The genes surrounding the *Pi3K21B* ortholog are orthologous to the genes at the same locus in *D. melanogaster* and local synteny is completely conserved, supported by results generated from *blastp* , so we conclude that LOC6503336 is the correct ortholog of *Pi3K21B* in *D. ananassae* ( Figure 1A ).

***Protein Model***

*Pi3K21B* in *D. ananassae* has four protein-coding isoforms ( *Pi3K21B* -PB, *Pi3K21B* -PC, *Pi3K21B* -PD, *Pi3K21B* -PE; Figure 1B ). *Pi3K21B* -PB has an identical coding sequence to *Pi3K21B* -PE, and *Pi3K21B* -PC has an identical coding sequence to *Pi3K21B* -PD. In *D. melanogaster* , mRNA isoforms *Pi3K21B* -RB and *Pi3K21B* -RE have two protein-coding CDSs, and *Pi3K21B* -RC and *Pi3K21B* -RD have one protein-coding CDS. Relative to the ortholog in *D. melanogaster* , the protein isoform count is conserved, but mRNA isoforms *Pi3K21B* -RC and *Pi3K21B* -RD have two CDSs in *D. melanogaster* . The sequence of *Pi3K21B* -PB in *D. ananassae* has 73.41% identity (E-value: 0.0) with the protein-coding isoform *Pi3K21B* -PB in *D. melanogaster* , as determined by *blastp* ( Figure 1C ). Coordinates of this curated gene model are stored by NCBI at GenBank/BankIt (accession BK064621 ). These data are also archived in the CaltechDATA repository (see “Extended Data” section below).

***Special characteristics of the protein model***

In *D. melanogaster* , *Pi3K21B* -RC and *Pi3K21B* -RD have one CDS, but in *D. ananassae* , they have two CDSs ( Figure 1D ). The presence of this intron is supported by the RNA-Seq of Adult Males, Transcripts and Coding Regions Predicted by TransDecoder, and a predicted splice junction with a score of 76. Therefore, we conclude that this CDS split in two as a result of the evolutionary distance between *D. melanogaster* and *D. ananassae* .

## Methods

Detailed methods including algorithms, database versions, and citations for the complete annotation process can be found in Rele et al. (2023). Briefly, students use the GEP instance of the UCSC Genome Browser v.435 ( https://gander.wustl.edu ; Kent WJ et al., 2002; Navarro Gonzalez et al., 2021) to examine the genomic neighborhood of their reference IIS gene in the *D. melanogaster* genome assembly (Aug. 2014; BDGP Release 6 + ISO1 MT/dm6). Students then retrieve the protein sequence for the *D. melanogaster* reference gene for a given isoform and run it using *tblastn* against their target *Drosophila* species genome assembly on the NCBI BLAST server ( https://blast.ncbi.nlm.nih.gov/Blast.cgi ; Altschul et al., 1990) to identify potential orthologs. To validate the potential ortholog, students compare the local genomic neighborhood of their potential ortholog with the genomic neighborhood of their reference gene in *D. melanogaster* . This local synteny analysis includes at minimum the two upstream and downstream genes relative to their putative ortholog. They also explore other sets of genomic evidence using multiple alignment tracks in the Genome Browser, including BLAT alignments of RefSeq Genes, Spaln alignment of *D. melanogaster* proteins, multiple gene prediction tracks (e.g., GeMoMa, Geneid, Augustus), and modENCODE RNA-Seq from the target species. Detailed explanation of how these lines of genomic evidenced are leveraged by students in gene model development are described in Rele et al. (2023). Genomic structure information (e.g., CDSs, intron-exon number and boundaries, number of isoforms) for the *D. melanogaster* reference gene is retrieved through the Gene Record Finder ( https://gander.wustl.edu/~wilson/dmelgenerecord/index.html ; Rele et al *.,* 2023). Approximate splice sites within the target gene are determined using *tblastn* using the CDSs from the *D. melanogaste* r reference gene. Coordinates of CDSs are then refined by examining aligned modENCODE RNA-Seq data, and by applying paradigms of molecular biology such as identifying canonical splice site sequences and ensuring the maintenance of an open reading frame across hypothesized splice sites. Students then confirm the biological validity of their target gene model using the Gene Model Checker ( https://gander.wustl.edu/~wilson/genechecker/index.html ; Rele et al., 2023), which compares the structure and translated sequence from their hypothesized target gene model against the *D. melanogaster* reference gene model. At least two independent models for a gene are generated by students under mentorship of their faculty course instructors. Those models are then reconciled by a third independent researcher mentored by the project leaders to produce the final model. Note: comparison of 5' and 3' UTR sequence information is not included in this GEP CURE protocol (Gruys et al., 2025).

## Data Availability

Description: A GFF, FASTA, and PEP of the model. Resource Type: Model. DOI: https://doi.org/10.22002/fb30s-g8p65
